# Basic Mechanism of Surface Topography Evolution in Electron Beam Based Additive Manufacturing

**DOI:** 10.3390/ma15144754

**Published:** 2022-07-07

**Authors:** Christoph Breuning, Julian Pistor, Matthias Markl, Carolin Körner

**Affiliations:** 1 Chair of Materials Science and Engineering for Metals, Friedrich-Alexander-Universität Erlangen-Nürnberg, Martensstr. 5, 91058 Erlangen, Germany; matthias.markl@fau.de (M.M.); carolin.koerner@fau.de (C.K.); 2 Joint Institute of Advanced Materials and Processes, Friedrich-Alexander-Universität Erlangen-Nürnberg, Dr. Mack Str. 81, 90762 Fürth, Germany; julian.pistor@fau.de

**Keywords:** additive manufacturing, electron beam, process modeling, heat transfer, processing window, topography

## Abstract

This study introduces and verifies a basic mechanism of surface topography evolution in electron beam additive manufacturing (E-PBF). A semi-analytical heat conduction model is used to examine the spatio-temporal evolution of the meltpool and segment the build surface according to the emerging persistent meltpool domains. Each persistent domain is directly compared with the corresponding melt surface, and exhibits a characteristic surface morphology and topography. The proposed underlying mechanism of topography evolution is based on different forms of material transport in each distinct persistent domain, driven by evaporation and thermocapillary convection along the temperature gradient of the emerging meltpool. This effect is shown to be responsible for the upper bound of the standard process window in E-PBF, where surface bulges form. Based on this mechanism, process strategies to prevent the formation of surface bulges for complex geometries are proposed.

## 1. Introduction

Additive manufacturing has experienced growing attention in both research and industry, as it offers the possibility for unique approaches for product design and material usage [1,2,3,4]. Several techniques exist to achieve a layerwise buildup of parts [5]. Powder-bed-based fusion processes (PBF) however have been proven to be the most promising technique to achieve fully dense (99.5%) high-performance parts [6,7]. Using a high-power laser (L-PBF) or electron beam (E-PBF), complex geometries can be realized by melting prealloyed metal powder. While the final objective of PBF is the fabrication of arbitrary complex geometries, the initial process development is typically carried out by melting standardized cuboid geometries [8]. In order to establish standardized material-specific process windows, where samples with the necessary degree of consolidation and even surfaces can be fabricated, stable process parameter combinations of beam power and velocity have to be identified. Samples fabricated below the low energy boundary with insufficient energy input experience insufficient consolidation due to the limited melt pool depth and residual porosity is present in the final parts [9,10,11]. In contrast, excessive energy input above the high energy boundary of the classic process window leads to pronounced material transport and results in an uneven melt surface topography [12,13,14]. Effects of thermocapillary convection [15,16,17], as well as evaporation effects [18,19] and the exerted recoil pressure of the beam [20], are considered responsible according to the literature. This effect is most commonly described for lens-shaped melt pools and is already commercially used to create defined surface structures according to the *Surfi-Sculpt*^®^ process. The transition of the melt pool regime from a lens-shaped to a persistent line-shaped melt pool was observed for higher beam powers and velocities [21,22]. The mechanism describing the material transport for a lens-shaped melt pool, however, is insufficient to explain the occurring material transport for specific process parameter combinations that result in pronounced surface bulges.

In this work, the spatio-temporal melt pool evolution is investigated as a function of process parameters to clarify the underlying correlation between the emerging melt pool behavior and the resulting surface topography. For this purpose, numerical simulations of the melt pool evolution are directly compared with experiments. Based on the results, suitable strategies are deduced to prevent the formation of surface bulges for simple as well as complex geometries.

## 2. Materials and Methods

### 2.1. Experimental

For the investigation of the melt pool evolution and the emerging surface topography, single-layer remelting experiments were performed without powder on priorly manufactured CMSX-4 substrate material, and samples were fabricated from the powder bed. The fabrication of the substrate was performed using an A2 E-PBF System (Arcam AB, Mölndal, Sweden) operating under EBM Control 5.2 using a Helium pressure of ${2\times 10}^{-3}$ mbar after initial evacuation to ${5\times 10}^{-5}$ mbar. Dense samples were achieved using the cross snake hatching strategy at a build temperature of 1030 ${}^{\circ }C$ with a line energy density of 0.9 J mm^−1^ and a line offset of 150 μm. For a detailed description of the E-PBF setup and the processing of CMSX-4 the reader is referred to previous publications [22,23,24,25]. The cuboid samples, with an edge length of 15 mm, were cut into slices of 3 mm thickness using a Brillant 220 device (ATM Qness GmbH, Mammelzen, Germany) and cleaned using an ultrasonic cleaner. For the single layer remelting experiments up to nine slices were positioned on an INCONEL^®^ 718 start plate and introduced into the E-PBF machine. For better heat insulation, the start plate was incorporated in the powder bed. The remelting took place at 1000–1020 ${}^{\circ }C$ according to the thermocouple at the bottom of the start plate. Due to severe heat loss of the small specimens, an additional heating step to reach the target temperature was added after remelting of each individual specimen. For more information about the remelting procedure the reader is referred to the recent publication by Rausch et al. [26]. To investigate the influence of the 90${}^{\circ }$ hatch rotation, 15 × 15 × 25 $mm^{3}$ samples were fabricated from the powder bed. Here, the respective process parameters are given in the images of the individual samples. For a better comparison of the energy input between process parameters, the respective Area Energy $E_{a}=\frac{P}{l_{o}\;\cdot \;v}$ is given, where *P* is the beam power, *v* the velocity and $l_{o}$ the line offset. For the analysis of the final surface topographies of each sample, laser scanning microscopy (LSM) was performed using a Lext OLS 4000 device (Olympus Europa SE & CO. KG, Hamburg, Germany) with 50 magnification.

### 2.2. Semi-Analytical Heat Conduction Model

A semi-analytical heat conduction model was used to calculate the spatio-temporal melt pool evolution. This model neglects effects of fluid convection, latent heat release, radiation, and vaporization, but enables the parallel evaluation of the temperature field and showed promising results in predicting the melt pool geometries [21,22]. The temperature *T* at time *t* is based on an analytical solution for the transient temperature response to a moving volumetric Gaussian heat source (Equation (1)) [21,27]:

(1) $$T\left(t,x,y,z\right)-T_{0}=\frac{2\eta P}{c\rho {\left(\pi /3\right)}^{3/2}}\int_{0}^{t}\frac{1}{\sqrt{\phi_{x}\phi_{y}\phi_{z}}}exp\left(-\frac{3x{\left(t^{\prime }\right)}^{2}}{\phi_{x}}-\frac{3y{\left(t^{\prime }\right)}^{2}}{\phi_{y}}-\frac{3z{\left(t^{\prime }\right)}^{2}}{\phi_{z}}\right)dt^{\prime }$$

with

(2) $$\phi_{i}=12\alpha \left(t-t^{\prime }\right)+\sigma_{i}^{2}\;for\;i=x,y,z$$

where $T_{0}$ describes the preheating temperature, *P* is the beam power, η the absorption coefficient, ρ the density, *c* the specific heat. The Gaussian beam shape is defined in each dimension by a beam width $\sigma_{i}$ and the thermal diffusivity α according to Equation (2).

The motion of the heat source is described by the coordinate system where x, y, z describe the distance from the point of interest to the transient location of the beam at time $t^{\prime }$. The piece-wise definition of the scan paths prohibits the analytical integration of Equation (1). Therefore a Gaussian quadrature scheme, proposed by B. Stump et al. [28] is used to numerically integrate the temperature at a given time and location. Material properties are assumed to be constant and uniform. The estimated material parameters adapted from Rausch et al. [26] are summarized in Table 1.

**Figure 1:**
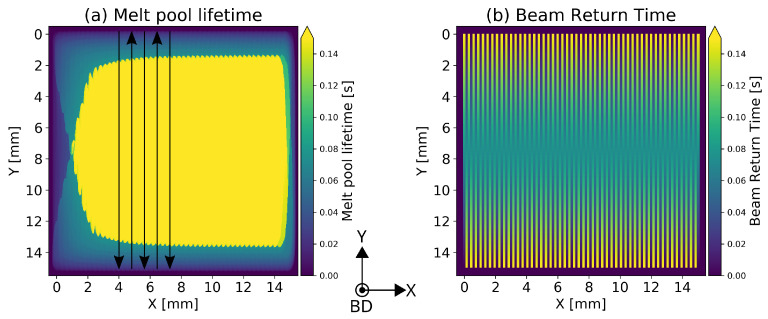
Approach for the determination of the persistent areas of the melt pool: (**a**) maximum melt pool life time at each position of the hatch; (**b**) local beam return times. Black arrows indicate beam movement along the scanning vectors according to the cross snake hatching strategy. Lateral movement of the beam from left to right.

The surface temperature distributions for each process parameter set is calculated to evaluate the melt pool evolution over the course of the hatch. In order to obtain continuous information about the melt pool evolution, the time step size was chosen such that the beam motion within one time step is limited to the length of one beam diameter per time step. The melt pool envelope of each time step was determined using the liquidus temperature. Previous research showed that melt pool geometries in the stationary state of the hatch can be classified as trailing or persistent. A melt pool is identified as persistent when a line shaped melt pool emerges and the melt pool is still liquid when the beam returns to the same position of the adjacent hatch line [21]. This persistence condition is fulfilled, when the beam return time is smaller than the local melt pool life time. The beam return time over the course of a single hatch line is linear, and the maximum return time depends on the lateral velocity $v_{lat}=\frac{l_{o}\;\cdot \;v}{l_{m}}$ of the beam, where $l_{m}$ is the hatch line length. In order to determine the spatial distribution of persistence formation, the maximum melt pool lifetime (Figure 1a) is compared to the return time of the beam (Figure 1b) at each position in the hatch.

## 3. Results

### 3.1. E-PBF Build Process

**Figure 2:**
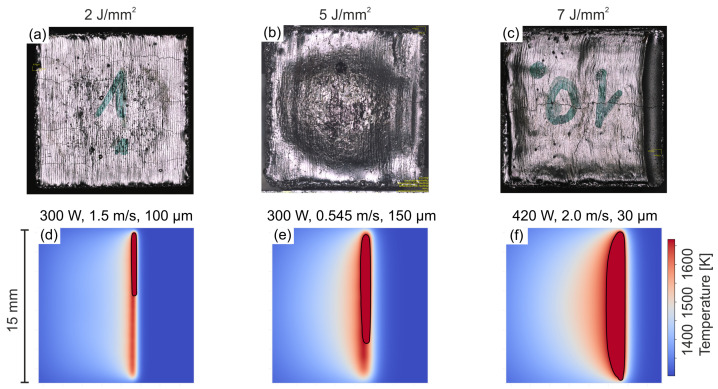
Experimentally observed surfaces (**a**–**c**) and calculated temperature fields (**d**–**f**) for parameters illustrating the continuous evolution of a trailing melt pool (**a**,**d**) to a fully persistent melt line (**c**,**f**). The intermediate condition (**b**,**e**) presents a persistent melt pool that is locally restricted to the center region. black lines in the temperature fields represent T_Liquidus_ of CMSX-4. Lateral melt direction from left to right.

Figure 2 shows the surface of the final melt layer of samples fabricated from powder bed for three different process parameters. The process parameters were selected to represent characteristic surface morphologies in the E-PBF process. Straight melt lines are visible according to the scan vectors in the sample with low-energy input in Figure 2a. On the surface of the sample with higher energy input, a local surface depression can be observed at the end of the hatch in the center of Figure 2b. With the emergence of a surface depression at the end of the hatch, the individual melt lines are no longer visible as straight melt lines, but are instead bent according to the shape of the surface depression. In case of the local surface depression in Figure 2b, straight melt lines can still be observed near the edges of the hatch. The depression spreads towards the edges of the samples with further increasing energy input, until the complete sample width is covered in Figure 2c. These observations are complimented by the calculated temperature fields in Figure 2, where the melt pool envelope is represented by the solid black line. In the case of the sample with low energy input a trailing, lens-shaped melt pool traverses the surface along the scanning vectors in Figure 2d. With higher energy input, distinct domains in the center of the hatch remain liquid when the beam returns in the subsequent line (Figure 2e). Further increasing the energy input leads to the formation of a line-shaped melt pool (Figure 2f), and the extension of the surface depression towards the turning points to cover the complete hatch. Consequently, no more straight hatch lines are observed on the surface near the turning points of the hatch.

### 3.2. Single Layer Remelting

**Figure 3:**
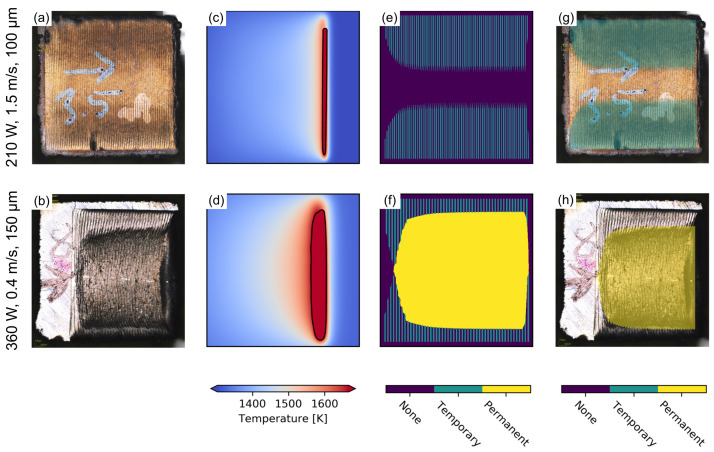
Experimentally observed surfaces (**a**,**b**) of single layer remelting experiments on CMSX-4 substrate with corresponding temperature fields (**c**,**d**) and calculated persistent regions (**e**,**f**). Superposition of simulation and experiment in (**g**,**h**). Lateral melt direction from left to right.

Figure 3 shows melt surfaces and corresponding temperature fields of single layer remelting experiments for two parameters with different energy input. Similar to Figure 2, distinct melt lines can be observed on the surface that are either aligned with the scan vectors in the trailing melt pool regime (Figure 3a,c), or are bent according to the emerging local surface depression for samples fabricated with higher energy input (Figure 3b,d). Based on the numerical simulations of the temperature field, the emerging persistent regions on the surface can be calculated according to the description in Section 2.2. In case of parameter combinations with small energy input, a trailing melt pool traverses the hatch. Near the turning points of the beam, however, persistent regions are identified in Figure 3e according to the definition in Section 2.2, which alternate sides with each subsequent hatch line. Since the melt pool is already solidified when the beam returns from the opposite side, these areas are only persistent on one side of the hatch and are defined as temporary persistent.

Over the course of the hatch, these areas expand further towards the center of the hatch until a stationary state is reached. In case of the parameter combination with higher energy input in Figure 3f, the temporary persistent regions on both sides exceed the center line and create an interleaving region in the center of the hatch. Within this region, the melt pool is still liquid when the beam returns from either turning point and is defined as permanent persistent. In this case, the evolution of the persistent regions over the course of the hatch is more pronounced compared to the parameter combination with lower energy input. In addition to the shape of the melt lines on the surface and the emerging surface depression at the end of the hatch, specific surface features can be observed in Figure 3a,b, that can be correlated with the calculated persistent regions. To correlate the observed features with temporary and permanent persistent regions, the results of both experiment and simulation are superimposed in Figure 3g,h. The surface of the sample with low energy input can be divided into distinct regions based on the surface color in Figure 3a. These regions agree with the determined temporary persistent regions. Both types of persistent regions can be further distinguished based on their respective surface morphology. While the change of the surface morphology is small in the temporary persistent regions, the induced change is more pronounced in the permanent persistent regions, and a clear difference of the surface morphology can be observe in Figure 3b.

#### Complex Geometry

**Figure 4:**
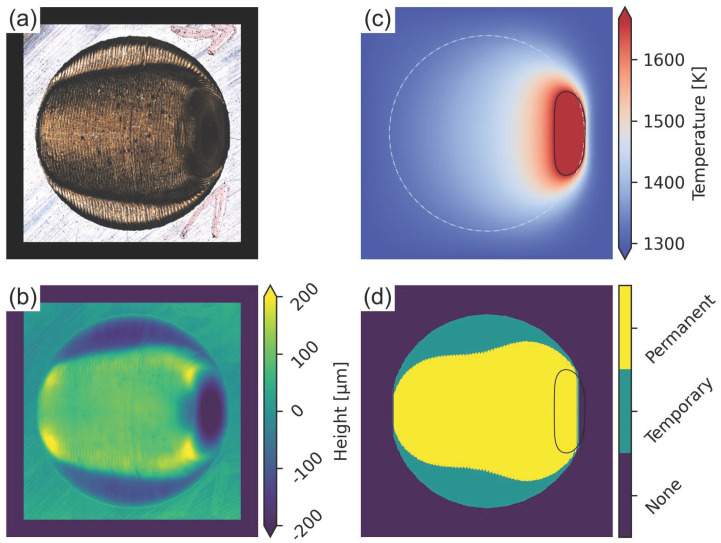
(**a**) Surface of a remelted single layer circle on CMSX-4 at 1000 ${}^{\circ }$C with a lateral melt direction from left to right. (**b**) corresponding surface topography according to laser scanning microscopy. (**c**) predicted temperature field of the circle geometry (light grey) and final melt pool (black) according to numerical simulation. (**d**) calculated persistent regions (temporary persistent regions are filled for better visibility) and final melt pool (black).

The numerical calculation of the melt pool lifetime enables the prediction of persistent regions and their correlation with the final surface topographies for complex geometries. For this purpose, a single layer remelting experiment on CMSX-4 substrate was conducted using a cylindrical cross section with variable scan lengths (Figure 4a). In this case, no Arcam Auto Functions were used to compensate for the scan length variation. The final surface topography according to laser scanning microscopy in Figure 4b can clearly be correlated with the predicted persistent domains in Figure 4d. While the temporary persistent regions near the turning points of the hatch exhibit a reduced height compared to the substrate surface, additional material is deposited in the permanent persistent domain in the center of the hatch. In addition, a small elevated ridge can be seen at the outer edges of the temporary persistent region. The calculated temperature field in Figure 4c, shows the last active melt pool, which is in good agreement with the experimentally observed surface depression in Figure 4b.

### 3.3. Hatch Rotation

**Figure 5:**
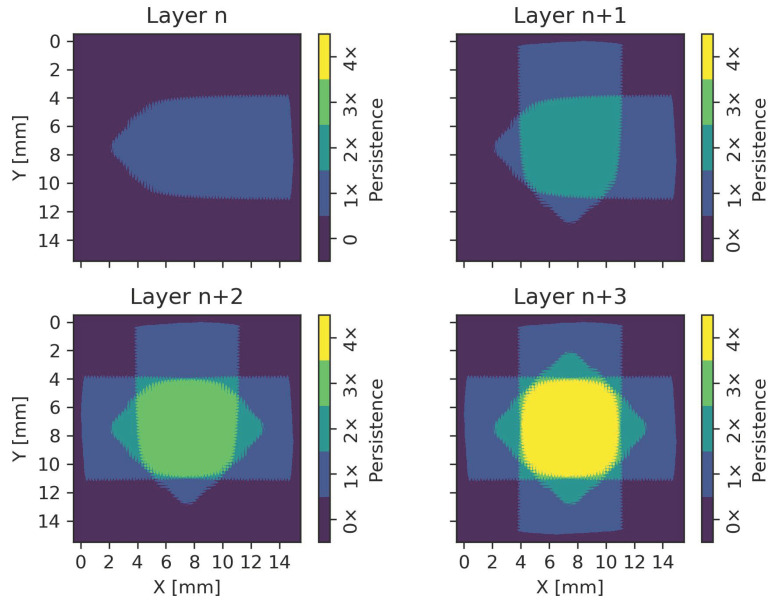
Superposition of permanent persistent regions due to a scan rotation of 90${}^{\circ }$ after each layer. The color represents the number of scan rotations where a specific region shows permanent persistence. Lateral melt direction from left to right for layer n. Lateral melt direction turning anti-clockwise for layer n + i.

In order to correlate the calculated persistent regions with the final surface topography of the E-PBF process, the scan rotation after each layer has to be considered. The application of a 90${}^{\circ }$ rotation after each layer results in a superposition of persistent regions in subsequent layers, which is illustrated in Figure 5. In this case, only permanent persistent regions are considered, as they are expected to have the largest influence on the surface topography. Layer n in Figure 5 represents the first layer with a lateral melt direction from the left to the right. The permanent persistent region in the centers emerges after a specific distance and expands further towards the turning points until the stationary state is reached. After an anti-clockwise rotation of 90${}^{\circ }$ in the subsequent layer n + 1, the melt pool progresses from the bottom towards the top, and the persistent regions of both layers cover the same region in the center of the hatch, as indicated in green. By further considering the two remaining rotations of layer n + 2 and layer n + 3, the final superposition of permanent persistent regions can be calculated. Regions where a persistent melt pool emerges for each of the 4 rotations are indicated in yellow in Figure 5.

**Figure 6:**
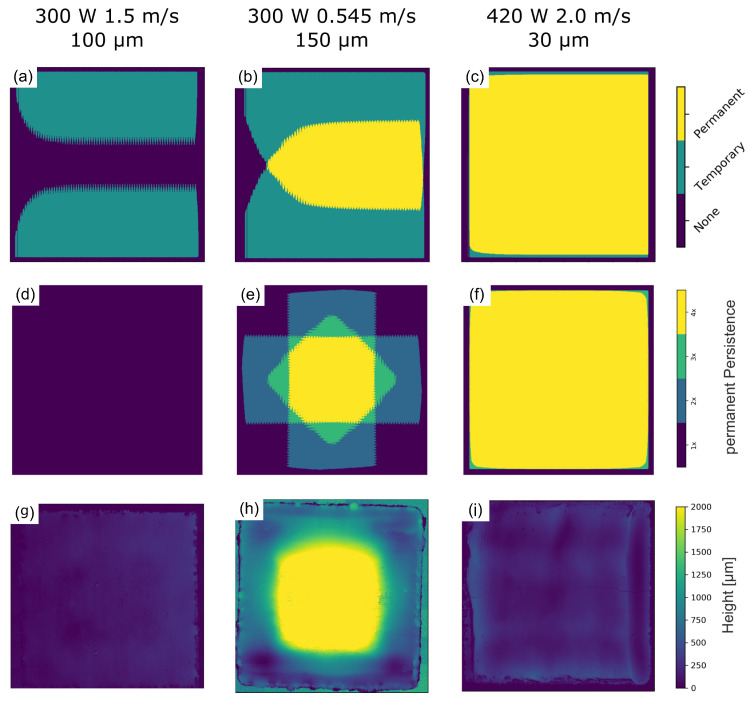
Persistence formation according to numerical simulation and the resulting surface topography for 500 consecutive layers with 90${}^{\circ }$ hatch rotation. (**a**–**c**) predicted persistence for one individual layer with lateral melt direction from left to right. (**d**–**f**) predicted permanent persistent regions according to superposition due to a 90${}^{\circ }$ scan rotation. (**g**–**j**) experimentally observed CMSX-4 surface topography according to laser scanning microscopy.

Figure 6a–c shows the calculated persistent regions for each parameter set of the E-PBF build process for one single layer. Temporary persistent regions are filled for better visibility. The superposition of permanent persistent areas for 4 subsequent layers in Figure 6d–f reveal distinct differences for each parameter set. The corresponding surface topographies determined by laser scanning microscopy are shown in Figure 6g–i. As expected, no permanent persistent regions emerge in the case of the low energy sample and an even surface topography is observed. The sample with higher energy input, in contrast, shows the emergence of a distinct permanent persistent region in the center of the hatch that also corresponds well with the observed surface depression at the end of the hatch in Figure 2b. The surface topography reveals a strong surface bulge of several millimeter in the center of the hatch in Figure 6h. The superposition of permanent persistent regions in Figure 6e shows a region in the center of the surface where a permanent persistent melt pool is present for all scan rotations. The permanent persistent region of the sample with the highest energy input stretches over the complete surface for each of the four subsequent scan rotations and an even topography without bulges can be observed in Figure 6i.

## 4. Discussion

The spatio-temporal evolution of the melt pool over the course of a hatch can be divided into distinct local melt pool regimes. Initial temporary persistent domains form at the turning points. During the transient phase of the hatch, the superposition of temperature fields from subsequent hatch lines then leads to cumulative heating, and the melt pool lifetime increases gradually until a quasi-stationary state is reached. Hence, temporary persistent domains extend further towards the center of the hatch until a constant extension is reached, as can be observed in Figure 3e. The evolution of the persistent domains in the transient phase, as well as the shape in the quasi-stationary state, are unique for each process parameter combination. By decreasing the beam return time, or increasing the melt pool lifetime, the temporary persistent domains extend further towards the center of the hatch until they eventually interleave and a permanent persistent melt pool emerges, as can be observed in Figure 3b. By further decreasing the beam return time or increasing the melt pool lifetime, the permanent persistent melt pool expands further to cover the complete surface.

As detailed in Section 3, each of the different melt pool regimes and persistent regions exhibits different surface morphologies and topographies. The different surface colors of the temporary persistent melt pool observed in Figure 3a can be attributed to the heterogeneous distribution of surface oxides that form at the end of the solidification and accumulate on the top surface as a result of different densities [29]. In case of the trailing melt pool in the center, spherical oxides are formed continuously and are randomly distributed along the scanning vectors. In the temporary persistent regions, however, these oxides are distributed along distinct lines at the rear end of the melt pool, making these regions distinguishable from the trailing melt pool region in the center of the hatch.

Local changes of the surface topography are the consequence of local material transport. Material transport is driven by the effects of recoil pressure on the melt pool due to evaporation effects [15,16,17,18,19,20]. Temperature gradients within the melt pool simultaneously lead to a a gradient of the surface tension, which decreases with a higher surface temperatures [16,17,30,31,32], and promotes material transport at the liquid-gas interface along the gradient of the surface tension. This phenomenon is well known as thermocapillary convection, or Marangoni convection [33]. The amount and direction of the combined material transport therefore depend on the surface temperature distribution and respective melt pool volume.

**Figure 7:**
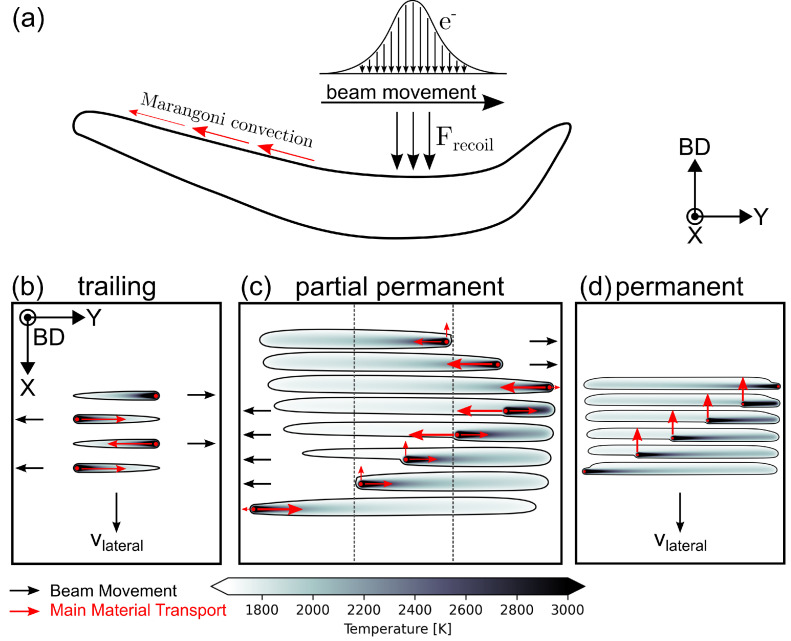
(**a**) Schematic illustration of material transport phenomena with respect to melt pool movement. (**b**) material transport and beam scanning direction for the trailing melt pool regime. (**c**) material transport and lateral movement for partial permanent persistent melt pool regime. (**d**) material transport for a permanent persistent melt pool regime.

In case of a trailing melt pool, a teardrop shaped melt pool follows the beam movement along the surface and the material transport phenomena are well studied [34,35]. While the surface temperature peaks at the beam position, the corresponding surface tension is lowest and results in a surface tension gradient that drives directed material transport from the front of the melt pool towards the tail of the melt pool, as indicated in Figure 7a,b. Recoil pressure acts at the beam position, where high temperatures promote evaporation and result in the displacement of material in each direction.

Since the melt pool volume and melt pool lifetime are small, material transport remains minor. By superimposing material displacement and beam movement for a single line, only material transport towards the tail and the sides of the melt pool remains, as all material displaced towards the front of the melt pool is reincorporated into the melt pool. The material transport towards the tail of the melt pool from one line, however, is counteracted by the subsequent line moving in the opposite direction, as detailed in Figure 7b and results in an even surface, as observed experimentally in Figure 3a. The material transported in opposite direction of lateral movement onto already solidified material, that is not reincorporated into the melt pool in the subsequent hatch lines, can be seen as straight rest lines on the surface in Figure 2a and Figure 3a [35].

In case of a permanent persistent melt pool, the same underlying principles governing material transport can be applied. In this case, however, the highest temperature gradient as well as surface tension gradient and therefore material transport, due to thermocapillary convection, arise in the direction opposite to the lateral melt pool propagation direction. As a result, material is transported towards the rear end of the melt pool. The superposition with the beam movement shows that material transport in this direction is not counteracted, and material is transferred from the liquid melt pool onto previously solidified material (Figure 7d), leaving characteristic lines in the shape of the melt pool on the surface, as observed in Figure 2c. The combination of material transport towards the rear end of the melt pool for each scan direction, and a high melt pool volume, results in major material transport. When the permanent persistent domain covers the complete sample width, material transport takes place homogeneously over the complete sample surface, and an even surface with a surface depression at the end of the hatch emerges (Figure 6f,i). The surface depression at the end of the hatch corresponds to the final permanent persistent melt pool, where material transport occurs and no subsequent hatch lines and material transport are able to compensate for the missing material [22].

Partial persistent melt pool regimes are defined by a combination of temporary and permanent persistent domains, as seen in in Figure 3b and Figure 4. In the permanent persistent domain of the hatch, a large melt pool forms and material transport is directed towards the rear end of the melt pool, resulting in a surface elevation. When the beam moves out of the permanent persistent region towards the turning points and back, the temperature and surface tension gradient is directed towards the permanent persistent region in the center of the hatch, and material transport occurs from the temporary persistent regions towards the center region. This process amplifies the surface elevation in the permanent persistent region, while surface depressions form in the temporary persistent domains, as seen in Figure 4b. When the beam moves from the turning point towards the center, initially material is transported towards the contour of the geometry and forms an elevated ridge, as seen in Figure 4b. Material that is deposited onto already solidified material at the side of the melt pool in the temporary persistent regions remains at its position and forms straight rest lines, that can be observed in Figure 3b and Figure 4a. The same effect can be observed for the material transport at the rear end of the permanent persistent melt pool domain, where rest lines form according to the shape of the melt pool, leaving behind the characteristic surface morphology. The surface depression at the end of the hatch in Figure 3b and Figure 4b again correspond to the final permanent persistent melt pool, where material transport occurs which cannot be compensated for.

The final surface topography of additively manufactured samples results from the superposition of material transport for each scan direction over the build height. If material transport is homogeneous, which is the case for a trailing melt pool (Figure 6a,d) and for a permanent persistent melt pool that covers the complete sample surface (Figure 6c,f), the superposition results in an even surface. In case of a partial persistent melt pool, however, material transport will be distinctly different for each region. While pronounced material transport occurs in the permanent persistent region in the center of the hatch, additional material is transferred from the temporary persistent regions to the center, leaving behind a surface depression. Due to the hatch rotation, the elevated persistent regions overlap in the center, where material is piled up in every layer of the build process. Consequently, surface bulges of several millimetres may build up in the center of the hatch, while the surface towards the edges stays leveled, as observed in Figure 6b,h. Uneven surface topographies lead to heterogeneous powder layer thicknesses in subsequent layers, and act as a source of consolidation defects [14]. Larger surface bulges can lead to significant process instabilities and can even lead to machine damage.

### Process Window and Prevention Strategies

**Figure 8:**
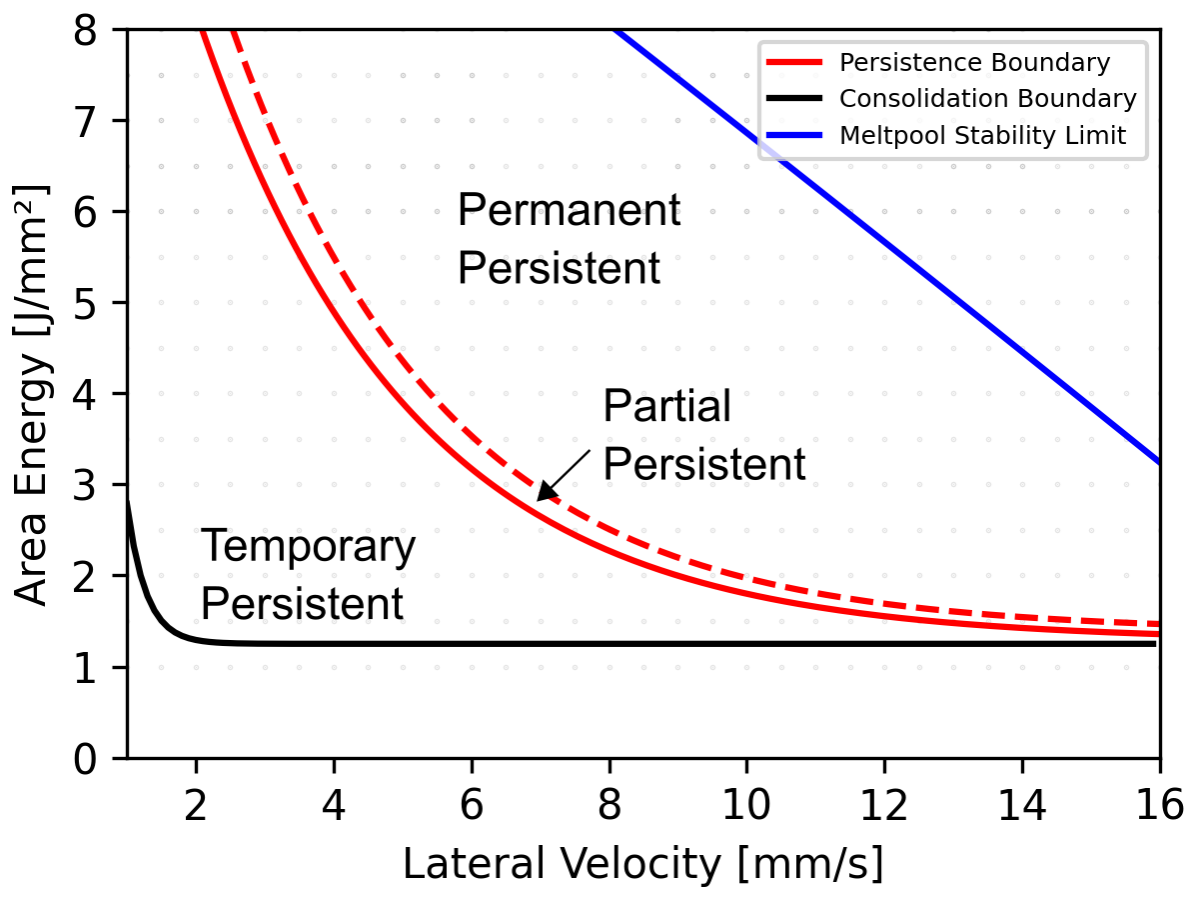
Simulated melt pool geometries in the stationary state of 512 process parameter combinations (gray) and respective processing boundaries of quadratic CMSX-4 samples with a side length of 15 mm and constant line offset of 100 μm, with a preheating temperature of 1020 ${}^{\circ }C$. The consolidation boundary (black) is defined by melt pools exceeding the effective layer thickness of (100 μm) [14]. The persistence boundary (red) is defined by the onset on a permanent persistent melt pool, which increases in size within the transition region until the permanent persistent region covers the complete sample (red stripes). The melt pool stability limit is reached when the ratio of lateral extension to melt pool depth reaches a critical limit of 4.7.

The transition from a trailing to a permanent persistent melt pool with increasing energy input and beam velocity may be responsible for the upper boundary of the classical process window, where fabricated samples show pronounced surface bulges [12]. However, as demonstrated in this work, samples with even surface topography can still be fabricated with parameter combinations exceeding the persistence boundary and the transition region, as shown in Figure 8, when the permanent persistent domain covers the complete sample width. Therefore, the classical process window can be further extended towards higher energy inputs and lateral velocities. In these cases, however, the melt pool geometry has to be closely controlled so as not to exceed the melt pool stability limit boundary [36]. This is especially important for complex geometries with changing line lengths and locally changing melt pool regimes.

The extent of the final material pile up for parameter combinations in the partial persistent melt pool regime, however, also depends on the actual melt pool geometry. While the necessary heat input for the formation of a permanent persistent melt pool decreases for higher lateral velocities, the melt pool volume also decreases. Additionally, the distance between the beam interaction area and the end of the melt pool increases, as the melt pool experiences a pronounced growth in lateral extension, which was already observed by Pistor et al. [22] and Breuning et al. [36]. Furthermore, the recoil pressure, as well as the surface tension gradient, are expected to be smaller as the peak temperatures on the melt pool surface relate to the line energy [19]. Regions in the process window with high lateral velocities are therefore not expected to be as prone to material transport as regions with lower lateral velocities. However, further work is required to validate the aforementioned hypothesis with focus on the hydrodynamics of different melt pool geometries.

Finally, based on the findings of this work, strategies to prevent surface bulges are based on establishing constant material transport conditions at each point of the hatch. This can be accomplished either by establishing a trailing or a permanent persistent regime, that covers the complete hatch area. In addition, leveraging lower beam return times in higher speed regions of the process window can also mitigate the risk of surface bulges and prevent subsequent process instabilities or even machine damage.

## 5. Conclusions

With the help of numerical simulations, the spatio-temporal evolution of the melt pool was classified into three different regimes as a function of processing parameters. Experimentally, each of the corresponding melt surfaces exhibit a characteristic surface morphology and topography, which emerge from the combination of material transport and beam movement. Material transport is driven by recoil pressure due to evaporation and thermocapillary convection along surface tension gradients. While homogeneous material transport occurs over the complete hatch surface for a trailing and permanent persistent melt pool, resulting in an even surface, a partial permanent persistent melt pool shows heterogeneous material transport. In this case, material is transported from the temporary persistent regions towards the permanent persistent domain, resulting in material accumulation and the creation of a surface depression of the temporary persistent regions. The final surface topography of an additively-manufactured part emerges from the superposition of material transport from all hatch directions of previous layers. While this effect was determined to be responsible for the upper limit of the processing window, dense samples with even surface morphologies can be fabricated above this limit with the onset of a permanent persistent melt pool that covers the complete sample width. Based on these findings, strategies to prevent surface bulges focus on establishing homogeneous material transport conditions, especially for complex geometries.

## Figures and Tables

**Table 1:** Material Properties for CMSX4.

Property	Value
Thermal diffusivity (m^2^ s^−1^)	$3.2\times 10^{-6}$
Density (kg m^−3^)	8193
Specific heat (J kg^−1^ K^−1^)	925
Absorption coefficient	0.85
Preheat temperature (K)	1273
Liquidus temperature (K)	1667

## Data Availability

Data supporting the findings of this study will be available upon reasonable request from the corresponding author.

## References

[1] Körner C. (2016). Additive manufacturing of metallic components by selective electron beam melting—A review. Int. Mater. Rev..

[2] Gu D.D., Meiners W., Wissenbach K., Poprawe R. (2012). Laser additive manufacturing of metallic components: Materials, processes and mechanisms. Int. Mater. Rev..

[3] Vayre B., Vignat F., Villeneuve F. (2012). Metallic additive manufacturing: State-of-the-art review and prospects. Mech. Ind..

[4] Wong K.V., Hernandez A. (2012). A Review of Additive Manufacturing. ISRN Mech. Eng..

[5] Frazier W.E. (2014). Metal Additive Manufacturing: A Review. J. Mater. Eng. Perform..

[6] Murr L.E., Martinez E., Amato K.N., Gaytan S.M., Hernandez J., Ramirez D.A., Shindo P.W., Medina F., Wicker R.B. (2012). Fabrication of Metal and Alloy Components by Additive Manufacturing: Examples of 3D Materials Science. J. Mater. Res. Technol..

[7] Gong X., Anderson T., Chou K. (2014). Review on powder-based electron beam additive manufacturing technology. Manuf. Rev..

[8] Markl M., Ammer R., Rüde U., Körner C. (2014). Numerical investigations on hatching process strategies for powder-bed-based additive manufacturing using an electron beam. Int. J. Adv. Manuf. Technol..

[9] Helmer H.E., Körner C., Singer R.F. (2014). Additive manufacturing of nickel-based superalloy Inconel 718 by selective electron beam melting: Processing window and microstructure. J. Mater. Res..

[10] Bauereiß A., Scharowsky T., Körner C. (2014). Defect generation and propagation mechanism during additive manufacturing by selective beam melting. J. Mater. Process. Technol..

[11] Tammas-Williams S., Zhao H., Léonard F., Derguti F., Todd I., Prangnell P.B. (2015). XCT analysis of the influence of melt strategies on defect population in Ti–6Al–4V components manufactured by Selective Electron Beam Melting. Mater. Charact..

[12] Juechter V., Scharowsky T., Singer R.F., Körner C. (2014). Processing window and evaporation phenomena for Ti–6Al–4V produced by selective electron beam melting. Acta Mater..

[13] Guo C., Ge W., Lin F. (2015). Effects of scanning parameters on material deposition during Electron Beam Selective Melting of Ti-6Al-4V powder. J. Mater. Process. Technol..

[14] Rausch A.M., Küng V.E., Pobel C., Markl M., Körner C. (2017). Predictive Simulation of Process Windows for Powder Bed Fusion Additive Manufacturing: Influence of the Powder Bulk Density. Materials.

[15] Amara E.H., Fabbro R., Hamadi F. (2006). Modeling of the melted bath movement induced by the vapor flow in deep penetration laser welding. J. Laser Appl..

[16] Pearson J.R.A. (1958). On convection cells induced by surface tension. J. Fluid Mech..

[17] Platten J.K., Villers D., Coninck J.D. (1990). An introduction to thermocapillary convection. Wetting Phenomena.

[18] Klassen A., Forster V.E., Juechter V., Körner C. (2017). Numerical simulation of multi-component evaporation during selective electron beam melting of TiAl. J. Mater. Process. Technol..

[19] Klassen A. (2018). Simulation of Evaporation Phenomena in Selective Electron Beam Melting. Ph.D. Thesis.

[20] Semak V., Matsunawa A. (1997). The role of recoil pressure in energy balance during laser materials processing. J. Phys. D Appl. Phys..

[21] Plotkowski A., Kirka M.M., Babu S.S. (2017). Verification and validation of a rapid heat transfer calculation methodology for transient melt pool solidification conditions in powder bed metal additive manufacturing. Addit. Manuf..

[22] Pistor J., Breuning C., Körner C. (2021). A single crystal process window for electron beam powder bed fusion additive manufacturing of a cmsx-4 type ni-based superalloy. Materials.

[23] Pistor J., Körner C. (2019). Formation of topologically closed packed phases within CMSX-4 single crystals produced by additive manufacturing. Mater. Lett. X.

[24] Körner C., Ramsperger M., Meid C., Bürger D., Wollgramm P., Bartsch M., Eggeler G. (2018). Microstructure and Mechanical Properties of CMSX-4 Single Crystals Prepared by Additive Manufacturing. Metall. Mater. Trans. A.

[25] Ramsperger M., Körner C. (2016). Selective electron beam melting of the single crystalline nickel-base superalloy CMSX-4^®^: From columnar grains to a single crystal. Superalloys 2016: Proceedings of the 13th International Symposium on Superalloys.

[26] Rausch A.M., Pistor J., Breuning C., Markl M., Körner C. (2021). New grain formation mechanisms during powder bed fusion. Materials.

[27] Nguyen N.T., Ohta A., Matsuoka K., Suzuki N., Maeda Y. (1999). Analytical solutions for transient temperature of semi-infinite body subjected to 3-D moving heat sources. Weld. J..

[28] Stump B., Plotkowski A. (2019). An adaptive integration scheme for heat conduction in additive manufacturing. Appl. Math. Model..

[29] Gruber H., Luchian C., Hryha E., Nyborg L. (2020). Effect of Powder Recycling on Defect Formation in Electron Beam Melted Alloy 718. Metall. Mater. Trans. A.

[30] Aune R.E., Battezzati L., Brooks R., Egry I., Fecht H.J., Garandet J.P., Hayashi M., Mills K.C., Passerone A., Quested P.N. Thermophysical properties of IN738LC, MM247LC and CMSX-4 in the liquid and high temperature solid phase. Proceedings of the International Symposium on Superalloys and Various Derivatives.

[31] Aune R., Battezzati L., Brooks R., Egry I., Fecht H.J., Garandet J.P., Mills K.C., Passerone A., Quested P.N., Ricci E. (2005). Measurement of thermophysical properties of liquid metallic alloys in a ground- and microgravity based research programme—The ThermoLab project. Microgravity Sci. Technol..

[32] Lee J., Shimoda W., Tanaka T. (2004). Surface tension and its temperature coefficient of liquid Sn-X (X=Ag, Cu) alloys. Mater. Trans..

[33] Marangoni C. (1871). Ueber die Ausbreitung der Tropfen einer Flüssigkeit auf der Oberfläche einer anderen. Ann. Der Phys..

[34] Yan W., Ge W., Qian Y., Lin S., Zhou B., Liu W.K., Lin F., Wagner G.J. (2017). Multi-physics modeling of single/multiple-track defect mechanisms in electron beam selective melting. Acta Mater..

[35] Shrestha S., Chou K. (2017). A build surface study of Powder-Bed Electron Beam Additive Manufacturing by 3D thermo-fluid simulation and white-light interferometry. Int. J. Mach. Tools Manuf..

[36] Breuning C., Arnold C., Markl M., Körner C. (2021). A multivariate meltpool stability criterion for fabrication of complex geometries in electron beam powder bed fusion. Addit. Manuf..

